# Depletion force optimization for high-purity gold nanotriangles prepared using different growth methods†

**DOI:** 10.1039/d3ra05955c

**Published:** 2023-11-02

**Authors:** Ryuichi Yamada, Ryusei Kimura, Shota Kuwahara

**Affiliations:** a Department of Chemistry, Faculty of Science, Toho University Funabashi Chiba 274-8510 Japan syouta.kuwahara@sci.toho-u.ac.jp

## Abstract

A homogeneous structural distribution in metal nanoparticle is commonly required for their application, and despite high-yield growth techniques, unavoidable structural heterogeneity remains a concern in metal nanoparticle synthesis. Gold nanotriangles (AuNTs) were synthesized using seed-mediated and seedless growth methods. Recent advancements in high-yield synthesis processes have enabled easy handling of AuNTs, which exhibit unique localized surface plasmon resonance characteristics due to their anisotropic triangular form. The flocculation and subsequent precipitation technique was used to purify AuNTs of different sizes synthesized using seed-mediated and seedless growth methods. The optimal conditions for obtaining high-purity AuNTs were explored by introducing a high concentration of cetyltrimethylammonium chloride. Additionally, the depletion force necessary for achieving high-purity AuNTs was calculated to reveal variations in the required depletion forces for AuNTs synthesized using different growth techniques. The alternations in the size distribution of AuNTs during the flocculation step were tracked using dynamic light scattering, and the surface charge of AuNTs synthesized through different growth methods was evaluated by *ζ*-potential. The high purity of the AuNTs produced using the seedless growth method required a larger depletion force than the seed-mediated grown AuNTs. The difference in the required depletion forces results from the difference in the electrostatic forces caused by the different growth methods.

## Abstract 2

The high purity of the gold nanotriangles produced using the seedless growth method required a larger depletion force than the seed-mediated grown gold nanoparticles.

## Graphical abstract

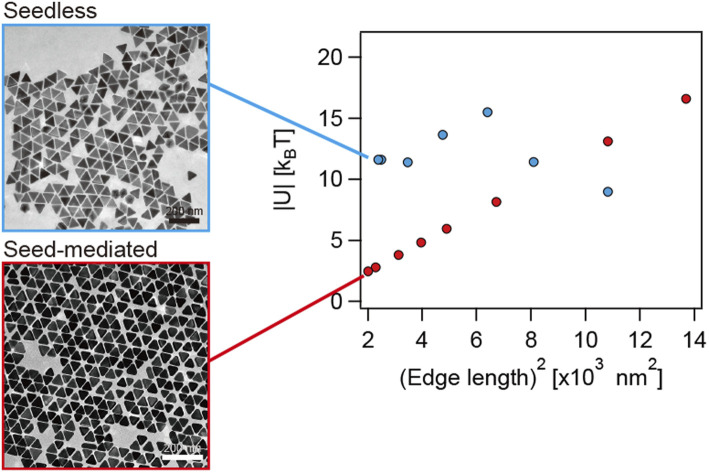

## Introduction

Metal nanoparticles exhibit intrinsic optical properties caused by the resonant oscillations of free electrons against externally induced electromagnetic waves, known as localized surface plasmon resonance (LSPR).^1,2^ The resonant energy of the LSPR depends on the density of free electrons and the distance between boundaries where electrons can move, hence the LSPR of metal nanoparticles can be modified by their constituent elements, size, and shape.^3,4^ Gold nanoparticles (AuNPs), in particular, have been extensively studied because of their chemical stability and wide-range modulation of LSPR in the visible light region. Various AuNP shapes (spheres,^5^ rods,^6–9^ cubes,^10,11^ stars,^12^*etc.*) have been synthesized using seed-mediated and seedless growth methods.^2,13–16^ The AuNP morphology is defined by thermodynamically or kinetically favored growth, and the growth speed of each facet of the nanoparticles is controlled by stabilizing the facet with constituents in the growth solution, *i.e.*, halide ions, Ag^+^, and surfactants.^17,18^

Gold nanotriangles (AuNTs) have also been synthesized using seed-mediated and seedless growth methods.^13,14,16,17,19–22^ Recent progress in the high-yield synthesis of AuNTs has made them easier to handle.^13,16,20,22^ Because of their anisotropic triangular form, AuNTs exhibit unique LSPR characteristics that cause hotspots at the corners, edges, and faces depending on the LSPR mode, which depends on the resonance against the incident light.^23–25^ The resonant energy of each LSPR mode in AuNTs may be modified by varying the length of the AuNT sides; the LSPR peak wavelength can modulate between 600 and 1300 nm.^22^ The anisotropic plate also exhibits unique assembled structures with edge-to-edge^13,16,20^ and face-to-face^26^ assemblies, resulting in a two-dimensional superstructure and a one-dimensional shuttle structure, respectively.

A homogeneous structure in metal nanoparticle is commonly required for their application, and despite high-yield growth techniques, unavoidable structural heterogeneity remains problematic in the synthesis of metal nanoparticles.^13,20^ To overcome this issue, various separation techniques, such as electrophoresis,^27,28^ filtration,^29,30^ centrifugation,^31^ and microfluidics,^32,33^ have been proposed. By modulating the differences in the size and/or surface charge of nanoparticles, such approaches have proven effective in obtaining metal nanoparticles with a high yield of homogeneous structures.

The sedimentation technique, which is commonly used for separating metal nanoparticles,^16,34,35^ sorts nanoparticles based on their stability in solution, with the increase in the entropy of the nanoparticles depending on the excluded volume effect. Depletion force between adjacent nanoparticles arise from an attractive osmotic pressure caused by a local concentration gradient, followed by the exclusion of the adsorbed surfactant micelles at the metal nanoparticle surfaces and subsequent replacement of the micelles with solvent molecules driven by the entropy of the system.^36^ A solution containing nanoparticles with varying shapes or sizes leading to significant variations in the depletion force, can be purified through subsequent aggregation under an elevated depletion force. This results in the formation of high-purity nanoparticles, which are found in the sediment within the solution. When AuNTs approach face-to-face followed by the formation of cylindrical aggregates, the exclusion volume of AuNTs is greater than that of nanospheres. The resulting shift in entropy stabilizes the aggregation of AuNTs, and the AuNTs selectively fall to the bottom of the solution under gravity. Huang *et al.* reported that the presence of sodium chloride as an additive induced the rapid precipitation of AuNTs,^20^ and using the sedimentation technique, they obtained AuNTs with a yield of >97%. However, required depletion forces to obtain high-purity AuNTs synthesized by different growth techniques weren't sufficiently discussed.

Herein, we present our findings on the depletion force optimization for achieving high-purity AuNTs *via* the flocculation and subsequent precipitation technique. Additionally, we discuss the distinctions in the depletion forces required to obtain high-purity AuNTs synthesized using different growth techniques, seed-mediated and seedless growth methods. We also examine the surface charge characteristics of each type of AuNTs. The optimized conditions for obtaining high-purity AuNTs were investigated by adding a constant volume of a high concentration of cetyltrimethylammonium chloride (CTAC) for seed-mediated growth and an optimal concentration of CTAC for seedless growth. The change in AuNT size distribution during the flocculation step was monitored using dynamic light scattering (DLS), and the surface charge of AuNTs synthesized by different growth methods was evaluated by *ζ*-potential. The seedless growth AuNTs (sg-AuNTs) were selectively precipitated with a depletion force of >11 *k*_B_*T*, which was higher than that required for precipitation of the seed-mediated growth AuNTs (smg-AuNTs).

## Experimental section

### Materials

Gold(iii) chloride trihydrate (HAuCl_4_·3H_2_O, ≥99%) was purchased from Merck-Sigma-Aldrich. CTAC (>95%) was purchased from Tokyo Chemical Industry Co., Ltd. Potassium iodide (KI), l-ascorbic acid (AA), 0.1 M sodium hydroxide solution (NaOH), and CV were purchased from Nacalai Tesque Inc. Sodium borohydride (NaBH_4_) was purchased from FUJIFILM Wako Pure Chemical Corp. All chemicals were used without further purification. Milli-Q water (resistivity 18.2 MΩ cm at 25 °C) was used in all experiments.

### Synthesis of AuNTs

#### Seed-mediated growth method

AuNTs were synthesized using the seed-mediated growth method described previously.^20^ Briefly, a seed solution was prepared by adding 450 μL of a freshly prepared, ice-cooled 0.01 M NaBH_4_ aqueous solution to a Au^3+^ solution consisting of 9.3 mL of 0.1 M CTAC and 100 μL of 0.02 M hydrogen tetrachloroaurate trihydrate. The obtained solution was vigorously stirred for 2 min. After 2 h of incubating at 30 °C in a water bath, the obtained seed solution was used for the subsequent synthesis of AuNTs.

The following two growth solutions were prepared for the synthesis of AuNTs. Solution (a): 100 μL of a 0.02 M HAuCl_4_·3H_2_O aqueous solution and 25 μL of a 0.01 M KI solution were sequentially added to 9.7 mL of a 0.1 M CTAC solution, followed by the addition of 185 μL of a 0.04 M AA solution for Au^3+^ reduction. Solution (b): 300 μL of a 0.02 M HAuCl_4_·3H_2_O aqueous solution and 90 μL of a 0.01 M KI solution were sequentially added to 29.1 mL of a 0.1 M CTAC solution, followed by the addition of 500 μL of a 0.04 M AA solution.

A diluted seed solution was prepared by adding 200 μL of the synthesized seed solution to 1.8 mL of a 0.1 M CTAC solution. The solution was added to solution (a) with manual stirring for 5 s. Then, an aliquot of this solution (0.1–1.4 mL) was immediately added to solution (b) and stirred for 10 s. The color of the solution changed from pink to purple, and then gradually to blue.

#### Seedless method

For typical growth procedure, 1.6 mL of a 0.1 M CTAC aqueous was added to 8.0 mL of Milli-Q water in a vial. Then, 75 μL of a 0.01 M KI solution and 80 μL of a 25.4 mM HAuCl_4_·3H_2_O aqueous solution were added sequentially.^13^ The obtained yellow solution changed to colorless after 20.3 μL of a 0.1 M NaOH solution and 80 μL of a 0.064 M AA solution were added sequentially. Finally, 10 μL of 0.1 M NaOH was injected and the vial was vigorously shaken by hand for 3 s. The solution was maintained at room temperature for 10 min. We modified the concentration and volume of CTAC, KI and AA to change the size distribution of sg-AuNTs.

### Purification of AuNTs

First, 1.0 mL of the AuNTs synthesis solution was mixed with 0.1 mL of a 25 wt% CTAC aqueous solution in a microtube. The AuNPs were allowed to precipitate for 18 h. The supernatant was then removed and the precipitated particles were redispersed in 1.0 mL of deionized water. Then, the solution was precipitated again with 0.1 mL of a 25 wt% CTAC aqueous solution. For the vial container instead of the microtube, 20 mL of the synthesized solution was mixed with 2.0 mL of a 25 wt% CTAC aqueous solution, the AuNPs were allowed to precipitate for 18 h, and the obtained supernatant was removed. Finally, the precipitate was redispersed in 5.0 mL of deionized water.

### Preparation of TEM samples

The purified AuNTs solution was centrifuged (4000 rpm (1000*g*), 15 min) and the supernatant was removed. After redispersion in 0.2 mL of deionized water, the obtained solution was centrifuged again, and the precipitated AuNTs were dispersed in 50 μL of deionized water. For the preparation of the transmission electron microscopy (TEM) samples, 50 μL of a solution was dropped onto a TEM grid on a Petri dish. The Petri dish was covered with Parafilm, covered with another dish, and incubated for 12 h.

### Characterization

TEM was performed using an electron microscope at 120 kV (Tecnai G2 F20 S-TWIN, FEI, Thermo Fisher Scientific, USA). The optical absorption spectra of the obtained samples were collected using an ultraviolet-visible-near infrared (UV-vis-NIR) spectrophotometer (UV-3600, SHIMADZU Corp., Japan). The size distribution and *ζ*-potential of the AuNTs were monitored using DLS and zeta potential measurement systems (ZETASIZER Nanoseries Nano-ZS, Malvern, UK).

## Results and discussion

### Synthesis and purification of seed-mediated growth AuNTs

**Scheme 1:**
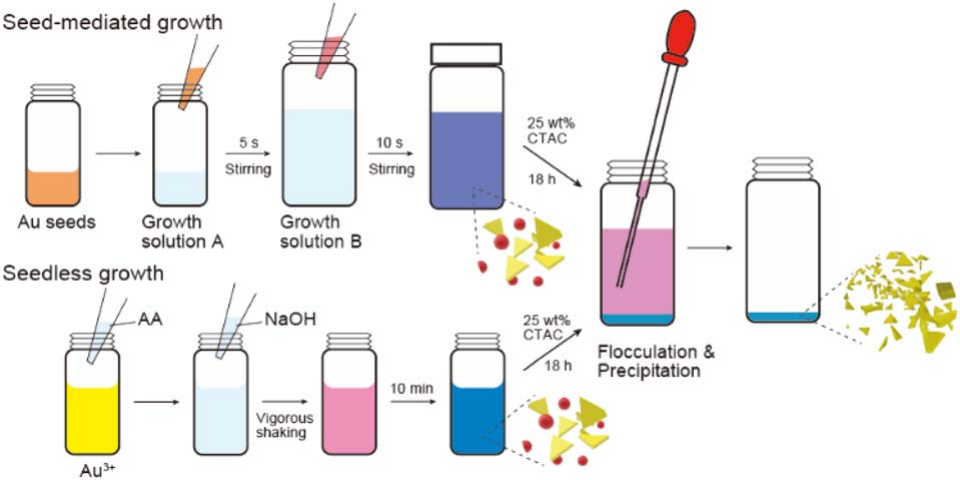
Schematic illustration of the growth and purification process of gold nanotriangles (AuNTs).

**Fig. 1:**
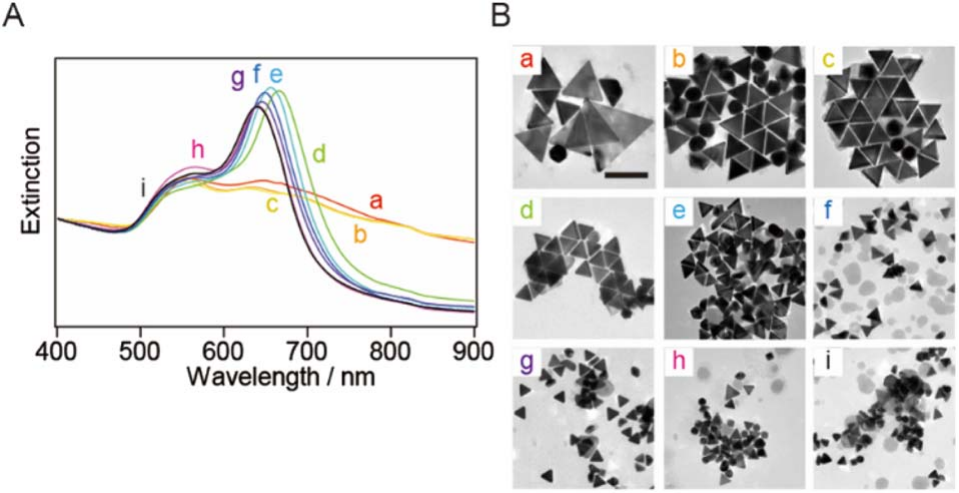
(A) Normalized extinction spectra and (B) typical TEM images of the seed-mediated growth AuNTs (smg-AuNTs) synthesized after different amounts of seed solution were transferred into the growth solution: (a) 0.1 mL, (b) 0.15 mL, (c) 0.2 mL, (d) 0.4 mL, (e) 0.6 mL, (f) 0.8 mL, (g) 1.0 mL, (h) 1.2 mL, and (i) 1.4 mL. The spectra were normalized at 400 nm. Scale bar: 200 nm.

smg-AuNTs were synthesized using the seed-mediated growth technique (Scheme 1).^20^ The size of the smg-AuNTs was controlled by altering the volumetric ratio of the seed solution to the growth solution. As the number of Au seeds increased, the number of consumed Au atoms per Au seed decreased, resulting in a decrease in the AuNT size.^16^ The size variation in the smg-AuNTs was confirmed using UV-vis spectroscopy and TEM analysis (Fig. 1). A blue shift in the plasmon band of the smg-AuNTs at ∼650 nm was observed when a large volume of Au seeds was added to the growth solution, and the edge lengths of the smg-AuNTs decreased from 133 to 45 nm. The smg-AuNTs synthesized using <200 μL of the Au seed solution had edge lengths >100 nm and settled easily as sediment, resulting in the low intensity of the AuNTs plasmon band in the extinction spectra of the supernatants. The purity of AuNTs before and after purification was evaluated by counting the structure of AuNPs by TEM (Fig. S1†). smg-AuNTs with edge lengths <50 nm showed lower purity (<40%), whereas smg-AuNTs with edge lengths of 50–100 nm showed purities of 40–60%.

**Fig. 2:**
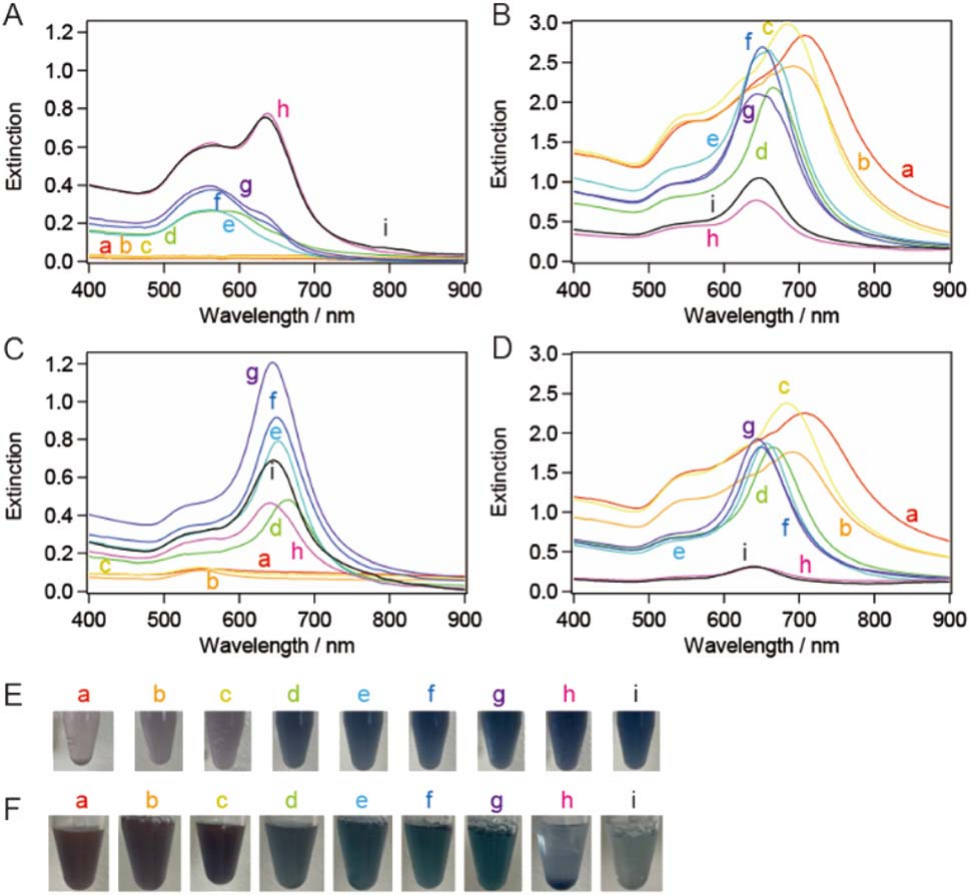
Extinction spectra of (A) the supernatant solutions and (B) the precipitate-redispersed solutions in the first purification step with a CTAC concentration of 94 mM. Extinction spectra of the (C) supernatant and (D) precipitate-redispersed solutions after the second purification step. The purification was performed on the sample solutions depicted in Fig. 1a–i. The AuNT sample solutions (E) before the purification (A) and (F) the precipitate-redispersed solutions after the second purification step (B).

The synthesized smg-AuNTs were purified using a flocculation and subsequent precipitation technique^34^ by adding a constant volume of a 25 wt% CTAC aqueous solution (0.1 mL of a 25 wt% CTAC aqueous solution in 1.0 mL of the AuNTs solution).^16^ The extinction spectra of the supernatant exhibited the plasmon band related to spherical AuNPs (∼550 nm), whereas those of the precipitate exhibited the plasmon band of AuNTs (Fig. 2A and B). As the size of the AuNTs decreased, the extinction intensity of the AuNTs in the supernatant increased, indicating that the number of AuNTs mixed in the supernatant increased when small AuNTs were purified *via* the precipitation technique.

To increase the purity of the AuNTs, a second purification step was performed. The extinction spectra of the precipitate obtained after the second purification exhibited the same spectral features in terms of peak positions and their full width at half maximum (FWHM) values as those obtained after the first purification. However, the spectra in the supernatant indicated that AuNTs were the main content (Fig. 2C and D) and the FWHM values changed in response to the purity of AuNTs. Thus, the AuNT size distribution after the first purification was maintained after the second purification. After the first purification step, AuNTs precipitated intensively at the bottom of the microtubes, whereas spherical AuNPs were mainly found in the supernatant. The solution color also had turned to purple, especially the larger AuNTs (Fig. 2E and F).

TEM was used to assess the purity of the AuNTs in the precipitate after the second purification, confirming the increased purity of the AuNTs to ∼80%, particularly in the size range of 60–80 nm (Table S1†). The solution containing AuNTs >100 nm also contained a significant amount of AuNPs >60 nm in size. This hindered the effective enhancement of the selective precipitation of AuNTs, primarily due to the amplified depletion force between AuNPs during the flocculation and subsequent precipitation. This phenomenon was driven by the increased curvature radius of the AuNPs' surface. The AuNTs <50 nm also showed a low improvement in AuNT purity in the obtained precipitate because the small difference in the depletion force between the AuNTs and AuNPs caused small changes in the flocculation manner between them.

**Fig. 3:**
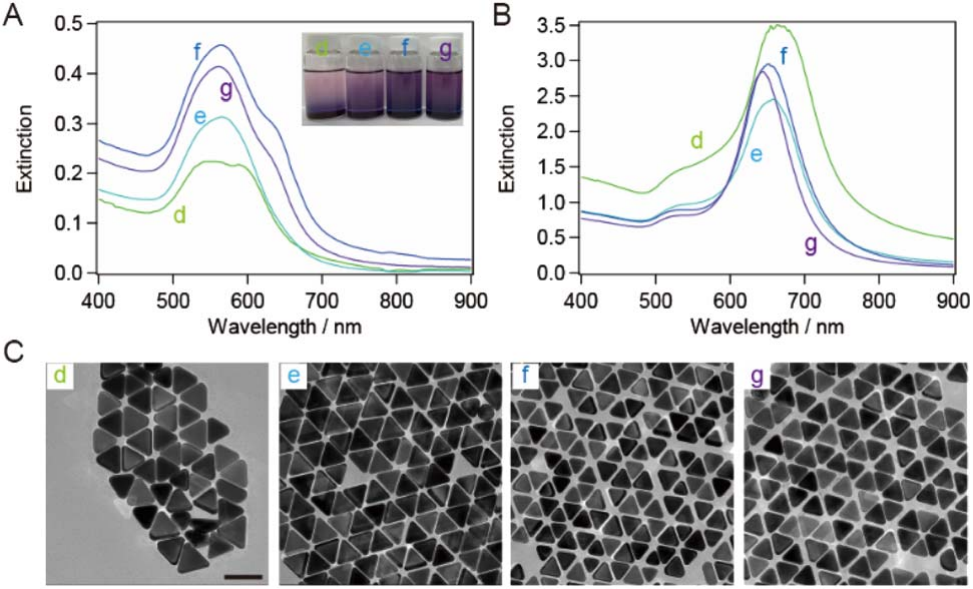
Extinction spectra of (A) the supernatant solutions and (B) the precipitate-redispersed solutions after purification in a vial. (C) Typical TEM images of the precipitates with average edge sizes of (d) 82 nm, (e) 70 nm, (f) 63 nm, and (g) 56 nm. Scale bar: 100 nm.

The bottom shape of the container used in the purification of AuNTs by flocculation and subsequent precipitation is reported to affect the selectivity of AuNTs contained in the precipitate.^37^ The AuNTs with sizes of 60–80 nm, which showed a purity of ∼80% in a microtube, were purified in a vial. The extinction spectra of the supernatant and precipitate in the vial (Fig. 3A and B) exhibited the identical spectral features concerning the peak positions and their FWHM values when compared to those in the microtube (Fig. 2A and B) although the FWHM values were influenced by the purity of AuNTs. As a result, the size distribution of the AuNTs remained consistent regardless of the container shape. However, the purity of the AuNTs in the precipitate obtained from the vial was higher than that of the microtube (Table S2†). In particular, the purities of the AuNTs with edge lengths of 70, 63, and 56 nm increased to >95%. Furthermore, the TEM observation (Fig. 3C) showed that the aggregation of AuNTs was a closed-packed self-assembled sheet, owing to the high purity of the AuNTs with a uniform size distribution.

### Synthesis and purification of seedless growth AuNTs

**Fig. 4:**
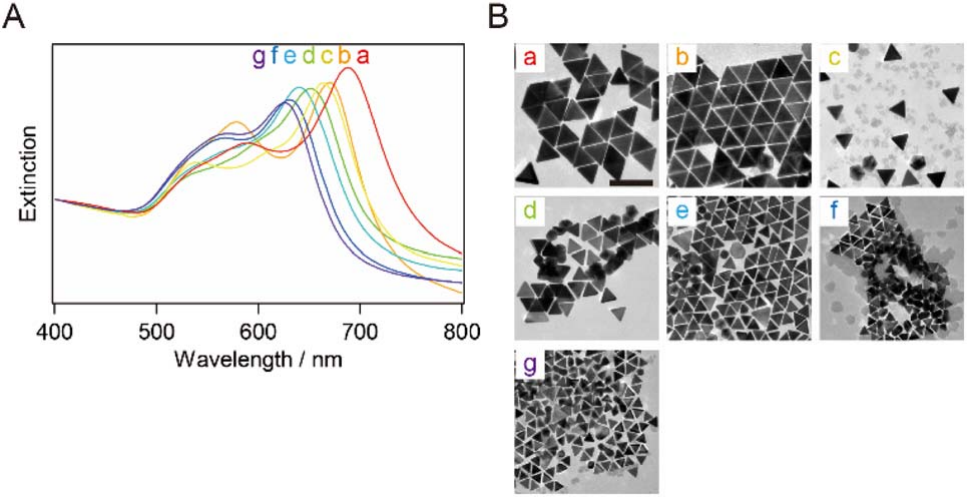
(A) Normalized extinction spectra and (B) typical TEM images of the synthesized seedless growth AuNTs (sg-AuNTs) with average sizes of (a) 104 nm, (b) 90 nm, (c) 80 nm, (d) 69 nm, (e) 59 nm, (f) 50 nm, and (g) 49 nm. The spectra were normalized at 400 nm. Scale bar: 200 nm.

sg-AuNTs were synthesized using the seedless method according to the previous paper (Scheme 1),^13^ and the size of the sg-AuNTs was controlled by altering the concentrations of constituents such as CTAC, KI, and AA in the seedless growth solution (Table S3†). The edge length of the obtained AuNTs was successfully increased from 49 to 104 nm, which was tuned to about the same size as the seed-mediated synthesized AuNTs. The change in the sg-AuNT size was confirmed using UV-vis spectroscopy and TEM analysis (Fig. 4). A blue shift of the AuNT plasmon band around 650 nm was observed when the size of the AuNTs decreased.

**Fig. 5:**
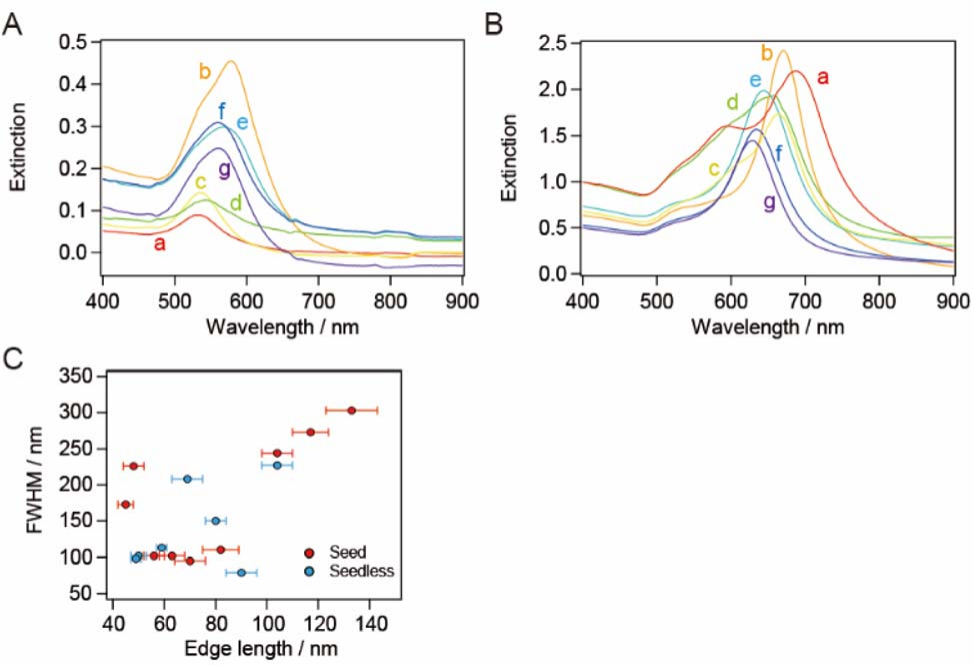
Extinction spectra of (A) the supernatant solutions and (B) the precipitate-redispersed solutions after the purification of the sg-AuNTs. The purification proceeded through the sample solutions depicted in Fig. 4. (C) Full width at half maximum (FWHM) as a function of the edge length of AuNTs synthesized using the seed-mediated (red) depicted in Fig. 2D and seedless (blue) growth methods depicted in (B), respectively. The error of FWHM is 2 nm.

The synthesized AuNTs were purified using the flocculation and subsequent precipitation technique. The volume of 25 wt% CTAC aqueous solution added to the solution was found to influence the generation of visibly separated precipitate in the microtube. The extinction spectra of the supernatant exhibited the plasmon band related to spherical AuNPs (∼550 nm), whereas those of the precipitate exhibited the plasmon band of AuNTs (Fig. 5A and B). Most of the AuNT solutions showed a purity of >90% (Table S3†). FWHM of the extinction spectrum of the purified sg-AuNT solution was compared with that of the smg-AuNTs (Fig. 5C). The FWHM values were determined using the extinction spectra depicted in Fig. 2D and 5B, with peaks corresponding to the LSPR of AuNTs around 600 nm. The FWHM values of the sg-AuNTs were almost the same as those of the smg-AuNTs except for the AuNT solutions with relatively low purity (edge length, 69 and 80 nm). These results demonstrate that the AuNTs were purified by flocculation and subsequent precipitation regardless of the growth method.

### Size distribution change of AuNTs monitored using DLS

**Fig. 6:**
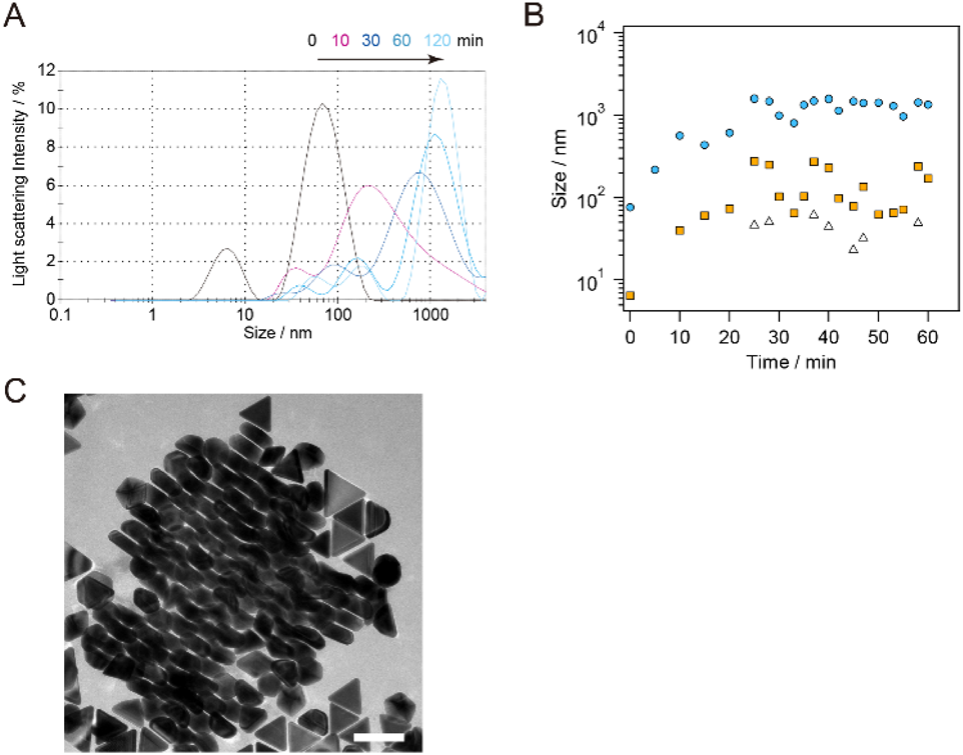
(A) The change in the size distribution of purified sg-AuNTs with an average edge length of 49 nm monitored by DLS with the addition of a 25 wt% CTAC aqueous solution (0.1 mL/1.0 mL). (B) The detected size peaks as a function of time: the average size of the largest (blue), middle (orange) and smallest (white) size distribution observed in DLS. (C) TEM image of a stacking structure found in the precipitate of AuNTs with an average edge length of 49 nm. Scale bar: 100 nm.

The change in the AuNT size during the flocculation step was monitored using DLS. sg-AuNTs with an edge length of 49 nm after purification (purity: 93%) were used for the monitoring by adding a 25 wt% CTAC aqueous solution (0.1/1.0 mL). The size distribution of the as-synthesized AuNTs exhibited a peak at 67.6 nm (distribution width (dw): 26.6 nm), which is consistent with the hydrodynamic diameter of circumscribed circle of the AuNTs used (*d* = 57 nm). The presence of co-existing spherical AuNPs (*d* = 35 nm) in the solution were minimal due to the purification steps, and it appears that the scattering probability of the co-existing AuNPs is lower than that of AuNTs. The addition of a high concentration of CTAC stimulated the aggregation of AuNTs, and the size distribution of the AuNTs increased to 569.0 nm (10 min, dw: 735.9 nm), 990.9 nm (30 min, dw: 696.4 nm), 1348 nm (60 min, dw: 750.2 nm), and 1408 nm (120 min, dw: 499.9 nm) (Fig. 6A). The increase in the size distribution of the AuNTs is attributed to the formation of AuNT aggregates with face-to-face stacking, as observed in the AuNT precipitate (Fig. 6C). Using an average thickness of 28.4 ± 3.0 nm for the AuNTs used, the number of stacked AuNTs was calculated as >50.

The peak observed in the DLS, >100 nm after 30 min, increased to 40.0 nm (10 min, dw: 10.9 nm), 102.8 nm (30 min, dw: 43.6 nm), 171.0 nm (60 min, dw: 64.3 nm), and 173.3 nm (120 min, dw: 53.5 nm), which is attributed to the change in the size of the co-existing spherical AuNPs with the CTAC stimulated aggregation remaining in the supernatant. The smallest size distribution observed in the DLS, which exhibited fluctuation around 50 nm, appears to be associated with individual AuNPs in the supernatant. The DLS results revealed that the rate of aggregation of the AuNTs was greater than that of the spherical AuNPs. Additionally, the small change in the size distribution caused by the aggregation of spherical AuNPs delayed the subsequent precipitation process of the AuNPs because of weak force for sedimentation compared to AuNTs, advancing the selective precipitation of AuNTs and increasing the purity of AuNTs.

**Fig. 7:**
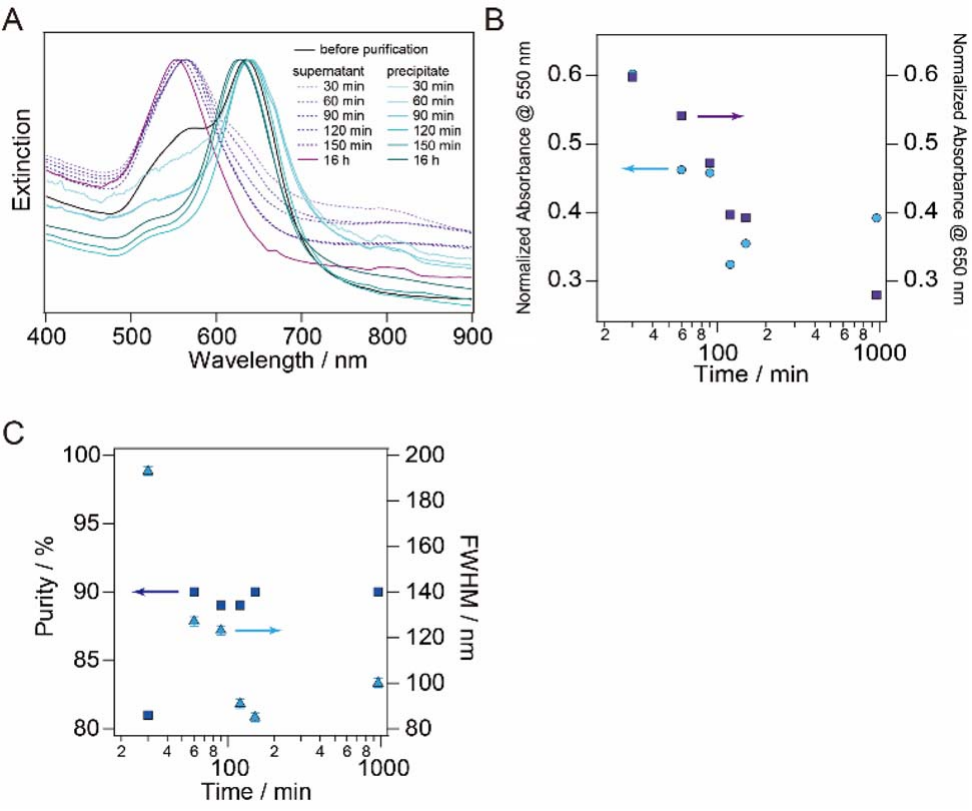
(A) Normalized extinction spectra of the time dependent precipitation of supernatant solutions (dot line) and precipitate-redispersed solutions (solid line). The black solid line represents the normalized extinction spectrum of a sample before the purification step. (B) The normalized extinction of the plasmon bands in the precipitate at 550 nm (blue circle) and the supernatant at 650 nm (purple square) as a function of time, plotted on a logarithmic scale on the horizontal axis. (C) The purity of AuNTs in the precipitates (purple square) and the FWHM of the plasmon band of the precipitate at ∼650 nm (blue triangle) as a function of time. Purity was assessed through TEM observation by counting 50 AuNPs, with the horizontal axis plotted on a logarithmic scale.

The time-dependence change in the extinction spectra of the supernatant and the precipitate-redispersed solutions during the flocculation step were monitored and are shown in Fig. 7A. As precipitation time increased, both the extinction of the plasmon bands at 550 nm (associated with spherical AuNPs) in the precipitate and that at 650 nm (associated with AuNTs) in the supernatant at 650 nm decreased, as shown in Fig. 7B. After 120 min of precipitation, the extinction of the plasmon bands associated with spherical AuNPs in the precipitate-redispersed solution remained consistently low, indicating a minimal and constant presence of spherical AuNPs in the precipitate. In contrast, the extinction of the plasmon bands related to AuNTs in the supernatant continued to decrease with longer precipitation times, resulting in an increased quantity of AuNTs including in the precipitation. The purity and FWHM values were ∼90% and ∼100, respectively, after 120 min (Fig. 7C), which corresponds to the extinction change observed in Fig. 7A. As indicated by the DLS results in Fig. 6, the increase in the size distribution of AuNTs precedes the precipitation of AuNTs dispersed in the sample solution, ultimately resulting in obtaining high-purity AuNTs in the precipitate.

### Depletion force for the purification of AuNTs

**Fig. 8:**
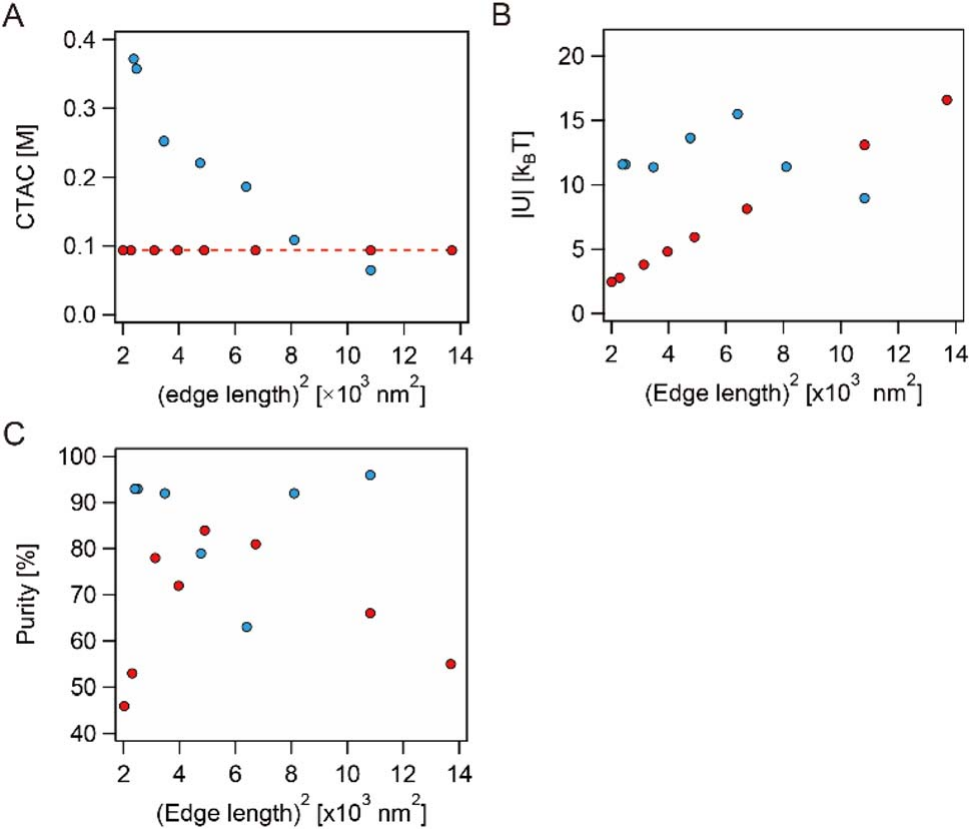
(A) The final CTAC concentration of the sample solutions prepared using the seed-mediated (red) and seedless (blue) growth methods. (B) Depletion force |*U*| of each sample solution prepared using the seed-mediated (red circle) and seedless (blue circle) growth methods as a function of (edge length)^2^ and (C) the purity of AuNTs in the precipitates of the seed-mediated (red cross) and seedless (blue cross) growth methods.

In this work, the final concentration of CTAC was constant for the smg-AuNTs, whereas the higher concentration of CTAC for the generation of visibly separated precipitate was used for the smaller sg-AuNTs (Fig. 8A). To reveal the optimized condition for obtaining high-purity AuNTs, the depletion interaction energy |*U*| was calculated using the equation proposed by Park *et al.*:^34^1
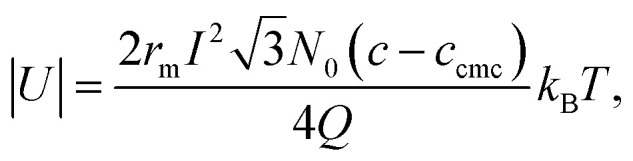
where *k*_B_ is the Boltzmann constant, *N*_0_ is Avogadro's number, *c* is the molar concentration of CTAC, *c*_cmc_ is the critical micelle concentration, *Q* is the aggregation number, *r*_m_ is the micellar radius, and *I* is the edge length of the AuNTs. The *c*_cmc_, *Q*, and *r*_m_ values of 0.001 M, 120, and 3 nm, respectively, were used according to previous reports.^16,38,39^ As shown in Fig. 8B, a |*U*| value equal to 11.6 *k*_B_*T* is needed for the selective precipitation of sg-AuNTs, whereas a value of 9.8 *k*_B_*T* is the optimal condition for purifying the smg-AuNTs, as previously reported.^16^ The sg-AuNTs with a purity <90% (Fig. 8B and C) were purified by changing the CTAC concentration and exhibited a |*U*| value of >12 *k*_B_*T*. This result indicates that the high depletion force induced flocculation of both spherical AuNPs and AuNTs, reducing the selectivity in the precipitate. In the case of the smg-AuNTs, the reduced purity of AuNTs with edge lengths <50 nm ((edge length)^2^ < 3 × 10^3^ nm^2^) is attributed to a reduction in the depletion force, which changed the precipitation speed, resulting in containing spherical AuNPs. Conversely, the reduced purity of AuNTs with an edge length >100 nm ((edge length)^2^ > 10^4^ nm^2^) is attributed to the flocculation of both spherical AuNPs and AuNTs.

The required depletion force for the sg-AuNTs was found to be larger by ∼2 *k*_B_*T* than that for the smg-AuNTs. The difference in the electrostatic interaction between the sg-AuNTs and smg-AuNTs was confirmed by the difference in the *ζ* potentials, which was +48.9 mV for the sg-AuNTs with an edge length of 49 nm and +32.5 mV for the smg-AuNTs with an edge length of 56 nm. Both AuNT solutions were measured after centrifugation and subsequent redispersion in water. The flocculation and subsequent precipitation of AuNPs were induced by the balance of depletion forces, van der Waals forces, and electrostatic interactions.^20^ The sg-AuNTs were expected to be more stable as a dispersion in a solution because of the charge repulsion force caused by the positive surface charge. The variation in repulsion forces is caused by the different number of adhered CTAC molecules on the AuNT surfaces in each growth mechanism. To overcome the electrostatic interaction force, a greater depletion force is required to flocculate the sg-AuNTs. Thus, the optimal conditions to obtain high-purity AuNTs using the flocculation and subsequent precipitation process will be affected by the growth manner of the AuNTs used.

## Conclusions

We have demonstrated the purification of AuNTs with different sizes synthesized utilizing the seed-mediated and seedless growth methods using the flocculation and subsequent precipitate technique. The size of the AuNTs increased from ∼40 to >100 nm, and each optimized condition for obtaining high-purity sg-AuNTs revealed that the required depletion force for the sg-AuNTs was larger than that for the smg-AuNTs. The difference in the required depletion force resulted from the variation in the electrostatic force produced by the different growth methods, which changes the number of adhered CTAC molecules on the AuNT surfaces. We have demonstrated the ability to obtain high-purity AuNTs *via* the flocculation and subsequent precipitation technique regardless of the difference in the growth mechanism of AuNTs. By obtaining high purity and generating a well-assembled structure of AuNTs, it is believed that AuNTs prepared using various growth techniques can be utilized for the optical application of their plasmonic properties.

## Author contributions

R. Y. and R. K. synthesized, purified, and characterized the gold nanotriangles. R. Y. and S. K. conducted the experiments. S. K. wrote the paper. All authors reviewed the manuscript.

## Conflicts of interest

There are no conflicts to declare.

## Supplementary Material

RA-013-D3RA05955C-s001
