# Supplementary material for: Depletion force optimization for high-purity gold nanotriangles prepared using different growth methods
Source: RSC Adv. 2023 Nov 2;13(46):32143–9. doi: 10.1039/d3ra05955c (PMC10620599; doi:10.1039/d3ra05955c)
Supplement: RA-013-D3RA05955C-s001 [file RA-013-D3RA05955C-s001.pdf]

Electronic Supplementary Material (ESI) for RSC Advances.
This journal is © The Royal Society of Chemistry 2023

Supporting Information for

Depletion Force Optimization for High-Purity Gold Nanotriangles Prepared Using Different Growth Methods

Ryuichi Yamada, Ryusei Kimura, and Shota Kuwahara*

Graduate School of Science, Toho University, 2-2-1 Miyama, Funabashi, Chiba 274-8510, Japan

Table S1. TEM analysis of the size and purity of seed-mediated growth AuNTs in the samples shown in Figures 1 and 2. 50 AuNTs were counted for the analysis, except for the seed solution of 1.0 mL (400 and 387 AuNTs were counted before and after the purification, respectively).

**Seed** /mL	λ_{max} /nm	**Edge** /nm	**Purity (as synthesized)** /%	**Purity (precipitate)** /%	**Diameter of spherical AuNPs** /nm
0.1	707	133±10	46	56	81±6
0.15	692	117±7	51	55	78±6
0.2	683	104±6	41	66	67±3
0.4	665	82±7	55	81	53±3
0.6	656	70±6	50	84	47±3
0.8	649	63±5	60	72	41±3
1.0	644	56±4	54	78	38±2
1.2	643	48±4	36	53	34±2
1.4	638	45±3	38	46	31±2

Table S2. TEM analysis of the purity of AuNTs for the samples shown in Figure 3. 50 AuNTs were counted for the analysis.

Seed /mL	Edge /nm	Purity (as synthesized) /%	Purity (precipitate) /%
0.4	82±7	55	81
0.6	70±6	50	84
0.8	63±5	60	72
1.0	56±4	54	78

Table S3. TEM analysis of the size and purity of seedless growth AuNTs in the samples shown in Figures 4 and 5. 50 AuNTs were counted for the analysis, except for the edge length of 49 nm (345 and 301 AuNTs were counted before and after the purification, respectively).

**Edge /nm**	**λ_{max} /nm**	**CTAC /M**	**KI /mM**	**AA /mM**	**Purity (as synthesized) /%**	**Purity (precipitate) /%**	**Diameter of spherical AuNPs /nm**
104±6	688	0.1	25	64	46	96	55±3
90±6	670	0.1	25	100	35	92	59±3
80±4	663	0.1	5	64	55	63	57±5
69±6	652	0.1	10	100	60	79	49±2
59±2	640	0.1	10	64	55	92	41±2
50±3	631	0.1	75	64	43	93	34±2
49±2	626	0.05	10	100	38	93	35±3

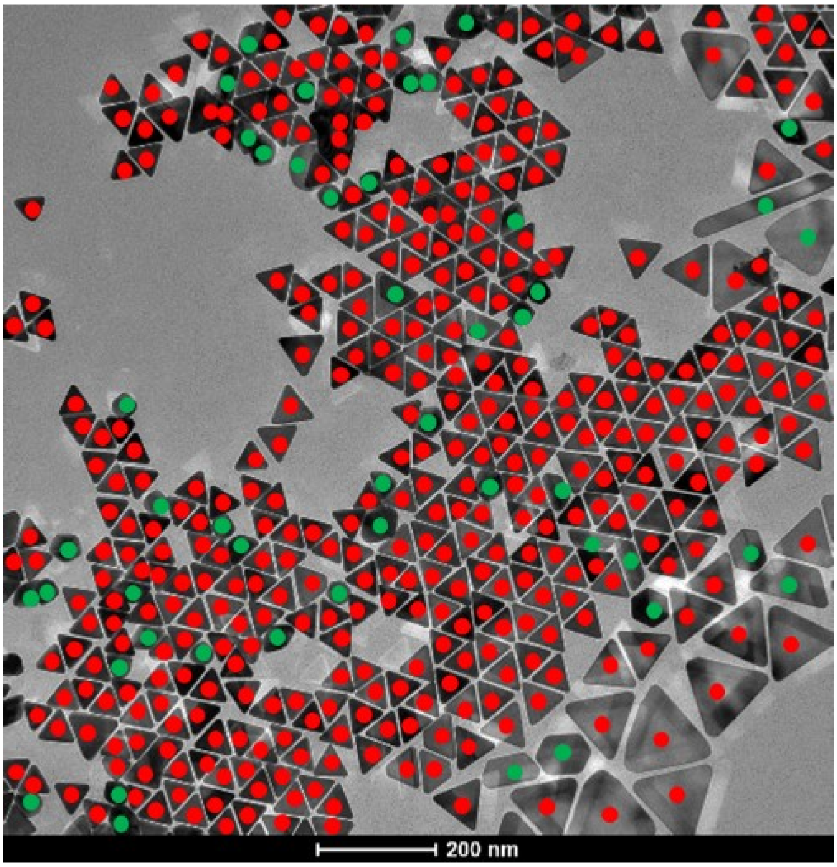

Figure S1. A large area TEM image for counting AuNTs in a sample. The particles were discriminated by their shape by color dots into AuNTs (red) other shapes of AuNPs (green).
